# Effects of Water Solutions on Extracting Green Tea Leaves

**DOI:** 10.1155/2013/368350

**Published:** 2013-08-07

**Authors:** Wen-Ying Huang, Yu-Ru Lin, Ruei-Fen Ho, Ho-Yen Liu, Yung-Sheng Lin

**Affiliations:** Department of Applied Cosmetology and Master Program of Cosmetic Science, Hungkuang University, Taichung, Taiwan

## Abstract

This study investigates the effects of water solutions on the antioxidant content of green tea leaf extracts. Green teas prepared with tap water and distilled water were compared with respect to four antioxidant assays: total phenol content, reducing power, DMPD assay, and trolox equivalent antioxidant capacity assay. The results indicate that green tea prepared with distilled water exhibits higher antioxidant activity than that made with tap water. The high performance liquid chromatography showed that major constituents of green tea were found in higher concentrations in tea made with distilled water than in that made with tap water. This could be due to less calcium fixation in leaves and small water clusters. Water solutions composed of less mineralisation are more effective in promoting the quality of green tea leaf extracts.

## 1. Introduction

Green tea is an infusion of the leaves of the *Camellia sinensis* plant, a member of the Theaceae family [1, 2]. As a popular drink favoured by Asians, in particular Chinese, green tea has received much global attention for its promotion of human health. A number of studies have shown that green tea possesses a wide range of biological activities [3], including antioxidant activity [4–9], anti-inflammatory activity [10], antimutagenic as well as anticarcinogenic activities [11–14], and neuroprotective effects [15]. The major functional constituents of green tea, catechins, account for 8–15% of the dry leaf weight [16].

A variety of catechins, which are members of the polyphenol family, are present in green tea leaves. The four most abundant green tea epicatechins are (–)-epigallocatechin gallate (EGCG), (–)-epicatechin gallate (ECG), (–)-epigallocatechin (EGC), and (–)-epicatechin (EC). In addition, four minor catechins are found in green tea leaves, including catechin (C), catechin gallate (CG), gallocatechin (GC), and gallocatechin gallate (GCG) [16, 17]. Catechins have been reported to possess strong antioxidant activity and are widely accepted as important antioxidants, which eliminate free radicals [6].

It is presumed that different tea-processing procedures and water components will result in different qualities of green tea [18–21]. Previous studies have demonstrated that the temperature [22–24], pH [25], organic solvent [26], pressure [27], soaking time [28], and elements of water [29, 30] affect the antioxidant activity of green tea. However, how the physics-based mechanism of the water solution affects tea extracts has rarely been discussed.

The aim of this study is to compare the effects of making green tea extracts with tap water versus distilled water on the antioxidant activities of the extracts. Comparisons are made using the results of four antioxidant activity assays and high performance liquid chromatography component analyses. Following the literature review, a possible physics-based mechanism to describe our observations is discussed. 

## 2. Materials and Methods

### 2.1. Chemicals and Reagents

The green tea leaves were from TenRen Tea Co., Ltd., Taipei, Taiwan. Gallic acid (GA), 2,2′-azino-bis(3-ethylbenzothiazoline-6-sulphonic acid) (ABTS), GC, EC, EGC, and EGCG were obtained from Sigma (USA). *N*,*N*-Dimethyl-*p*-phenylenediamine dihydrochloride (DMPD) and iron (III) chloride were purchased from Riedel-de Haen (Germany). Folin-Ciocalteu reagent was from Fluka (Germany). Methanol, acetonitrile, and acetic acid were purchased from Merck (Darmstadt, Germany).

### 2.2. Processing of Green Tea

Instead of a brewing process [7, 31], green tea solutions were prepared using tap water and distilled water at 25°C. The former was taken from the suburban area of the City of Greater Taichung, Taiwan. Distilled water (<1 **μ**S/cm) was purified using a Millipore Alpha-Q Luton ultrapure water system such that both the organic and inorganic impurities were removed. Two water samples were divided into 25 mL aliquots in 50 mL polypropylene centrifuge tubes. Aqueous extracts were prepared by soaking a 31.25 mg infusion of ground up and homogenized dry green tea leaves in 25 mL water at 25°C for 20 min. After centrifugation (1250 rpm), the green tea supernatant was collected for further examination; five replicates were made for statistical analysis.

### 2.3. Total Phenol Content

The total phenol content of green tea was measured according to Gutfinger's method [32]. Each green tea solution was mixed with 250 *μ*L Folin-Ciocalteu reagent for 1 min and 50 *μ*L 2% Na_2_CO_3_ for 5 min. The absorbance at 655 nm (Sunrise ELISA Plate Reader, Tecan, Austria) denoted the total phenol content, and it increased with the content of phenols.

### 2.4. Reducing Power

As described in a previous report [33], the reducing power of green tea was determined using a mixture of 250 *μ*L phosphate buffer (200 mM, pH 6.6), 250 *μ*L potassium ferricyanide (1% by weight), and 500 *μ*L green tea. The mixture was then incubated at 50°C for 20 min. Trichloroacetic acid (250 *μ*L, 10% by weight) was mixed with distilled water (250 *μ*L) and FeCl_3_ (750 *μ*L, 0.1% by weight), allowed to react for 30 min, and the absorbance at 700 nm was measured using a spectrophotometer. Higher absorbance indicated greater reducing power.

### 2.5. DMPD Assay

Antioxidant activities of green tea were measured according to Schlesier's method [34]. In principle, colorless DMPD will form a stable purple free radical cation, DMPD^+^•, in the presence of a suitable oxidant, such as FeCl_3_. Purple DMPD^+^• is scavenged and decolorized upon addition of an antioxidant. Equal volumes of 24 mM DMPD, 2.4 mM FeCl_3_, and 0.6 M acetic acid were mixed together for 5 min to generate DMPD^+^•, and the absorbance at 495 nm was measured. The antioxidant activity was inferred as the ability to scavenge free radicals, and a decrease in absorbance was inferred as increasing scavenging power, defined as follows:

(1) DMPD+•  scavenging  activity (%)  =  [1−(A2A1)]×100%,

where A1 denotes the absorbance of total free DMPD^+^• radicals and A2 denotes the absorbance following addition of the antioxidant. 

### 2.6. Trolox Equivalent Antioxidant Capacity Assay

The trolox equivalent antioxidant capacity (TEAC) assay was carried out using the procedure described by Erkan's method [35]. In brief, ABTS^+^• was produced by reacting 7 mM ABTS with 2.45 mM potassium persulfate in the dark at 4°C for 12 h. A 30 *μ*L antioxidant aliquot was added to 2 mL ABTS^+^• radical solution, allowed to react for 10 min, and the absorbance measured at 734 nm.

As stated previously, the antioxidant capacity was inferred as the ability to scavenge free radicals, expressed as follows:

(2) ABTS+•  scavenging  activity (%)  =  [1−(A4A3)]×100%,

where A3 represents the absorbance of total free ABTS^+^• radicals, and A4 represents the absorbance following addition of the antioxidant.

### 2.7. High Performance Liquid Chromatography

The extract of green tea was mixed with an internal standard solution (0.5 mg gallic acid diluted to 25 mL with 70% methanol) at a ratio of 1 : 1. The samples were spiked with various concentrations of stock solutions and then analyzed.

A stock solution was prepared by dissolving four marker substances (GC, 1.0 mg; EGC, 5.0 mg; EGCG, 2.5 mg; and EC, 10.0 mg) in 1 mL of 70% methanol; the solution was then stored in a refrigerator. Stock solutions were diluted with 70% methanol into a series of standard solutions (GC: 0.78, 1.04, 1.25, 1.56, 2.08, and 3.13 *μ*g/mL; EGC: 2.50, 6.25, 7.81, 10.41, 15.63, and 31.25 *μ*g/mL; EGCG: 2.60, 3.13, 3.91, 5.21, and 7.81 *μ*g/mL; EC: 2.08, 4.17, 4.63, 5, 6.25, and 8.33 *μ*g/mL). A 20 *μ*L aliquot of each solution was injected and analyzed two times using HPLC, and the standard curves were plotted as peak areas versus concentrations. Recovery was determined by comparing the amount of marker substances added to the marker substances found. The limits of detection were based on a signal to noise (S/N) ratio of 3 : 1 as a minimum.

HPLC was performed on an Agilent 1220 series system. Satisfactory separation of the markers was obtained using a reversed-phase column (Cosmosil 5C18-AR II, 5 *μ*m, 25 cm × 4.6 mm I.D., Nacalai Tesque, Kyoto, Japan) at 280 nm, an elution flow rate of 0.8 mL/min, and a linear solvent gradient of A-B (A, 10 mM KH_2_PO_4_ (pH 4.0); B, CH_3_CN, CH_3_OH, and H_2_O at a ratio of 1.5: 2.5:  1, respectively, [v/v/v]) as follows: 0 min, 20% B; 5 min, 20% B; 15 min, 30% B; 45 min, 60% B.

### 2.8. Statistical Analysis

The paired *t*-test method was used to evaluate differences between groups. A value of *P* < 0.05 was considered statistically significant (*), and *P* < 0.01 was highly significant (**).

## 3. Results and Discussion

### 3.1. Total Phenol Content

**Figure 1:**
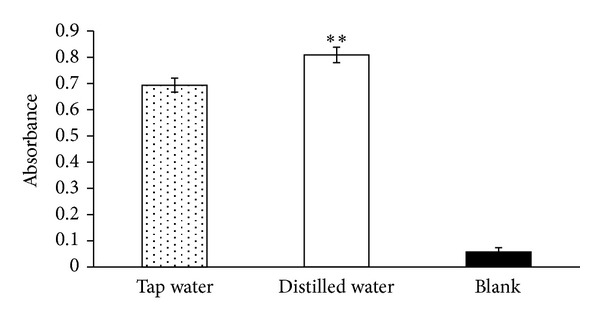
Effects of water solution on the total phenol content of green tea.

Figure 1 shows the differences in A_655_ between the tea solutions. Indicating the total amount of phenol, the A_655_ for the control (no green tea extract), green tea made with distilled water, and green tea made with tap water were 0.06 ± 0.02, 0.81 ± 0.03, and 0.69 ± 0.03, respectively. Phenols are regarded as significant constituents of green tea because of their free-radical scavenging ability.

### 3.2. Reducing Power

**Figure 2:**
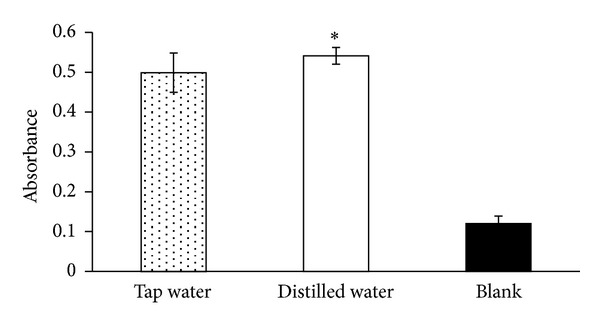
Effects of water solution on the reducing power of green tea.

The reducing power of a compound serves as an indicator of its antioxidant activity [33].  Figure 2 shows the reducing power (absorbance at 700 nm) of the 3 groups tested. The A_700_ for the control, green tea made with distilled water, and green tea made with tap water were 0.11 ± 0.02, 0.53 ± 0.03, and 0.47 ± 0.06, respectively.

### 3.3. DMPD Assay

**Figure 3:**
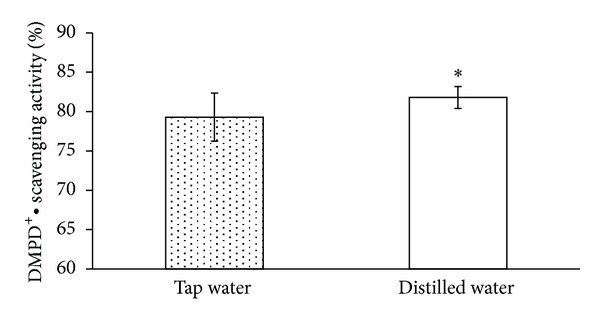
Effects of water solution on DMPD^+^• scavenging activity in green tea.

Figure 3 shows that the inhibition of DMPD^+^• radical cations by green tea was 84.04 ± 3.10% when made with distilled water and 82.15 ± 2.46% when made with tap water. Compared to tea made with tap water, there was a 1.89% improvement in DMPD^+^• scavenging activity in the tea made with distilled water. Thus, the distilled water was able to extract more antioxidant substances from the green tea leaves as determined by DMPD^+^• scavenging activity.

### 3.4. Trolox Equivalent Antioxidant Capacity Assay

**Figure 4:**
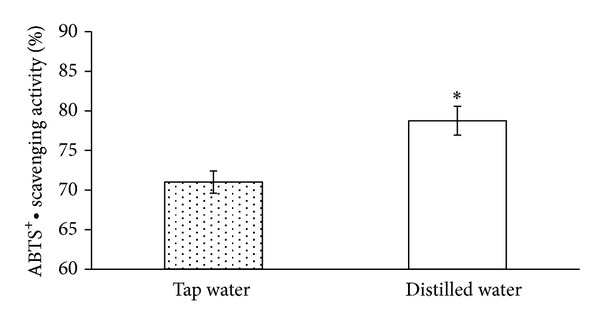
Effects of water solutions on ABTS^+^• scavenging activity in green tea.

Figure 4 indicates that ABTS^+^• radicals were inhibited by 78.75 ± 1.81% and 71.01 ± 1.41% in green tea made using distilled water and tap water, respectively. Results of the TEAC assay demonstrate that green tea made with distilled water has superior ABTS^+^• scavenging activity (7.74% increase) compared to green tea made with tap water. These results suggest that distilled water has a clear advantage over tap water in terms of its ability to extract more antioxidants into the tea solution.

### 3.5. High Performance Liquid Chromatography

Calibration curves were prepared by plotting the peak-area ratios (using gallic acid as an internal standard) against the corresponding concentrations. Using linear regression to analyze data in the concentration range of interest, the detection limits were between 0.078 and 1.25 *μ*g/mL (S/N = 3) for the components.

**Figure 5:**
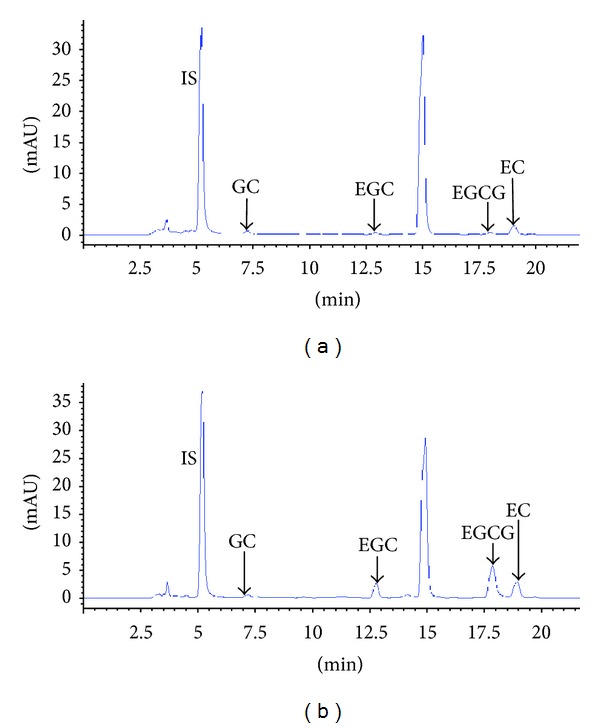
High performance liquid chromatography spectra of green tea prepared with different water solutions. (a) Tap water and (b) distilled water (IS = gallic acid, GC = gallocatechin, EGC = (–)-epigallocatechin, EGCG = (–)-epigallocatechin gallate, and EC = (–)-epicatechin).

By substituting the peak-area ratios of individual peaks acquired using HPLC, the contents of individual components in the green tea were determined. The average amounts of 4 constituents in teas made using tap water and distilled water, respectively, were (mg/g ± SD): GC, 4.43 ± 0.94 and 3.35 ± 0.53; EGC, 5.86 ± 1.78 and 21.03 ± 0.47; EGCG, 1.43 ± 0.07 and 12.36 ± 0.03; EC, 5.52 ± 0.21 and 10.26 ± 0.18. Of these 4 constituents, EGC, EGCG, and EC show higher amounts in the distilled water sample than its tap water counterpart, as shown in Figure 5.

### 3.6. Ion Concentration

The extraction efficiency of green tea leaves depends on the presence of electrolytes. Therefore, the ions present in water were further determined by the inductively coupled plasma mass spectrometry (SCIEX Elan 5000, Perkin Elmer, Überlingen, Germany).  Table 1 shows the cation contents in two waters of this study. There is a higher cation concentration in tap water than distilled water. Corresponding to previous reports [36], the higher the mineralisation, the lower the green tea leaves extraction yields of organic matter. The reason may be also attributed to that calcium is taken up by leaves from highly mineralised water and assumed to be complexed with pectins in cell walls. The formation of complex will retard extraction of green tea leaves thus explaining the decrease in extraction yield. Besides, tea polyphenols which are the leading functional component may combine with ions such as Ca^2+^ and Mg^2+^ to be partially retained in tea residue [37].

Except calcium fixation in leaves, the water structure may also affect extraction efficiency. Water structures have been studied for over 100 years in various research fields, including physics, chemistry, and physical chemistry [38]. Many theoretical and experimental approaches for studying the structure of water have been well developed (e.g., statistical thermodynamics, molecular dynamics simulation, infrared spectrum, Raman spectrum, X-ray diffraction, neutron diffraction, and NMR) [39–41]. Electrolytes have been shown to affect the median number of water clusters and the cluster size [39, 42]. This observation supports the idea that water organized into smaller clusters can extract more functional constituents of green tea leaves and hence enhances the antioxidant activity of green tea. The rate-determining step in tea leaf infusion was determined to be the diffusion of solutes through the leaf matrix to the surface [43]. Smaller water clusters provide larger surface areas, thus increasing contact opportunities between water molecules and green tea leaves and allowing the extraction of more solutes from the leaves. In addition, the interaction between the sample and the extracting solvent is enhanced due to the increased number of water clusters, resulting in a highly efficient extraction. In this study, cations increase the median water cluster size [39] and decrease extraction of green tea leaves.

## 4. Conclusions

This study investigated the effects of water conditions on the efficiency of constituent extraction from green tea leaves. The increase in polyphenol extraction in green tea prepared with distilled water increases its antioxidant activity. Our results revealed that distilled water may be a good choice for extracting compounds probably due to its less calcium fixation in leaves and smaller water cluster size, thus enhancing the antioxidant activity of green tea made with distilled water. These results provide an alternative application of distilled water in the processing of certain foods.

## Figures and Tables

**Table 1:** Cation compositions of tap water and distilled water.

Water	Cation concentration (ppb)				
	Ca^2+^	Al^3+^	Na^+^	Mg^2+^	K^+^
Tap water	21690	13.33	10120	6698	1361
Distilled water	115.2	0.465	9.035	2.159	0.941
